# Clinical efficacy and safety of pembrolizumab and nivolumab in frontline treatment for classical Hodgkin lymphoma: systematic review and meta-analysis of clinical trials

**DOI:** 10.3389/fonc.2026.1865991

**Published:** 2026-06-26

**Authors:** Artem Oganesyan, Andrew Gregory, Mark Gregory, Deivid Badalian, Asatur Chakmanyan, Lusine Harutyunyan, Vahe Khachatryan, Abhay Aradhya, Aheed Javaid, Yervand Hakobyan, Christian J Fidler, Pierluigi Porcu

**Affiliations:** 1 Department of Internal Medicine, Jefferson Abington Hospital, Abington, PA, United States; 2 Thomas Jefferson University, Philadelphia, PA, United States; 3 Department of Internal Medicine, University of Massachusetts Chan, Worcester, MA, United States; 4 Wayne State University School of Medicine, Detroit, MI, United States; 5 Department of Adult Hematology, Yeolyan Hematology and Oncology Center, Yerevan, Armenia; 6 Department of Hematology and Transfusion Medicine; National Institute of Health, Yerevan, Armenia; 7 Department of Internal Medicine, Desert Regional Medical Center, Palm Springs, CA, United States; 8 Department of Internal Medicine, Flushing Hospital Medical Center, New York, NY, United States; 9 Asplundh Cancer Pavillion, Sidney Kimmel Comprehensive Cancer Center, Willow Grove, PA, United States; 10 Division of Hematology and Bone Marrow Transplantation, Markey Cancer Center, University of Kentucky, Lexington, KY, United States

**Keywords:** Hodgkin lymphoma, immune checkpoint inhibitors, immunotherapy, nivolumab, PD-1, pembrolizumab

## Abstract

**Introduction:**

Treatment of classical Hodgkin lymphoma (cHL) underwent substantial advances throughout the last decades, where PD-1 inhibitors showed improved clinical outcomes, leading to their approval for relapsed and refractory disease. Recent studies showed benefit in utilizing these agents in first-line settings. Recent studies showed benefit in utilizing these agents in first-line settings. This meta-analysis investigates clinical efficacy and safety of pembrolizumab and nivolumab for frontline cHL treatment.

**Methods:**

A comprehensive literature search was conducted through PubMed, EBSCO Host, and CENTRAL using relevant keywords. Full-text publications in the English language of clinical trials were selected. Complete response rate (CRR), overall response rate (ORR), 2-year overall survival (OS), 2-year progression-free survival (PFS), and grade ≥3 adverse events (AEs) were evaluated for pooled analysis.

**Results:**

Nine trials, including 880 patients with previously untreated cHL (21% with early-stage disease) with a median age of 34 years and a median follow-up of 31.2 months, were analyzed. Pooled data in all treatments revealed ORR 86% (95% confidence interval (CI), 59-96%), CRR 57% (95% CI, 62-97%), 2-year PFS 90% (95% CI, 70-97%), and 2-year OS 97% (95% CI, 91%-99%). Forty-one percent of patients (95% CI, 21-64%) experienced grade ≥3 AEs, and 17% (95% CI, 6-38%) grade ≥3 immune-related AEs in all treatment groups. AVD with pembrolizumab or nivolumab showed comparable efficacy (2-year PFS: 97% (95% CI, 88-99%) vs 92% (95% CI, 89-94%), 2-year OS: 98% (95% CI, 88%-100%) vs 99% (95% CI, 99-100%) and safety (grade ≥3 AEs: 36% vs 33%), respectively. Nivolumab with brentuximab vedotin had worse outcomes (2-year PFS 80%, OS 87%, and grade ≥3 AEs 79%), likely due to an older study population (median age 71.5 years).

**Conclusions:**

Adoption of nivolumab or pembrolizumab combinations in frontline treatment for cHL is a valid strategy for advanced disease or patients who are unfit or not interested in intensive cytotoxic chemotherapy regimens.

## Introduction

Classical Hodgkin lymphoma (cHL) is a unique B-cell malignancy, in which a small number of neoplastic Hodgkin and Reed–Sternberg (HRS) cells reside within a dense and active non-malignant immune cell milieu (1). It makes up about 90–95% of all Hodgkin lymphoma cases and typically presents with cervical or mediastinal lymphadenopathy, often accompanied by constitutional “B symptoms”, such as fever, night sweats, and weight loss, which reflects its systemic and inflammatory nature.

Globally, there are around 83,000 new cases yearly, with the highest incidence of 2–3 cases per 100,000 population annually in high-income countries (2, 3). The distribution of cHL is bimodal with young (15–35 years old) and older adults (>60 years old) being primarily affected. Additionally, cHL usually occurs in individuals with a family history of this disease, EBV infection, or an immunodeficient condition (4).

Several key mechanisms allow the formation of the tumor microenvironment, the promotion of immune suppression, and tumor survival in cHL. In particular, activation of JAK/STAT and NF-κB pathways through overexpression of CD30 on HRS cells, EBV viral-driven survival signaling, as well as immune evasion via overexpression of programmed cell death ligand PD-L1 and PD-L2 (related to 9p24.1 amplification) are the crucial elements of the disease pathophysiology (5–7). This improved understanding of disease biology led to more mechanism-directed therapeutic options, such as anti-CD30 antibody-drug conjugate brentuximab vedotin (BV) and PD-1 inhibitors (nivolumab and pembrolizumab).

Treatment of cHL underwent substantial advances in the past decades. Historically, first-line treatment was ABVD (doxorubicin (Adriamycin), bleomycin, vinblastine, dacarbazine) in combination with radiotherapy to the site involved, achieving cure rates of 80-90%, especially in early-stage disease (8). Although a later developed escalated BEACOPP (bleomycin, etoposide, doxorubicin, cyclophosphamide, oncovin (vincristine), procarbazine, prednisone) led to improved disease control; however, high-toxicity rates (cytopenias, infections, osteonecrosis) limited widespread adoption of this regimen (9). Incorporation of BV further shaped the treatment strategy, leading to cure rates exceeding 90% in newly diagnosed patients, even with advanced disease (10, 11). The ECHELON-1 trial demonstrated substantial improvements with BV+AVD regimen compared to ABVD in progression-free survival (PFS) (82.3% vs 74.5%, hazard ratio (HR): 0.68; p=0.001) and overall survival (OS) (93.5% vs 88.8%; HR: 0.62; p=0.011) among over 600 patients with advanced cHL at 7 years of follow up (10, 11). The main limitation with the BV+AVD regimen was the risk of neutropenia, requiring G-CSF prophylaxis, and peripheral neuropathy. The German Hodgkin Study Group tried to address the side effects of escalated BEACOPP with the HD21 trial, where PET-guided BrECADD (BV, etoposide, cyclophosphamide, doxorubicin, dacarbazine, and dexamethasone) was more effective and better tolerated than escalated BEACOPP (12, 13). These results became a base of European Medicines Agency (EMA) approval for adult patients with previously untreated CD30-positive stage IIB with risk factors, stage III or IV cHL.

More recently, the S1826 trial, involving 994 patients, demonstrated that nivolumab combination with AVD (N+AVD) resulted in significantly better PFS (92% vs 83%, HR 0.45; 95% CI, 0.30–0.65) and a safety profile when compared to BV+AVD for frontline settings, making it a new standard of care for newly diagnosed advanced stage cHL (14). Importantly, this study supported the US Food and Drug Administration (FDA) approval for N+AVD regiment for adult and pediatric patients aged 12 years and older with previously untreated, Stage III or IV cHL in March of 2026 (15).

Nonetheless, these advancements bring new questions that have not been answered yet. It is unclear whether PD-1 inhibitors can be equally effective for different stages of disease (i.e., early-stage vs. advanced-stage) and whether the efficacy of these agents varies depending on histological subtype. It is also uncertain what the optimal combination of treatment regimen is and whether it should be individualized depending on patient characteristics.

Hereby, we performed a meta-analysis of clinical trials in order to provide better data on clinical efficacy and safety of most commonly utilized PD-1 inhibitors (nivolumab and pembrolizumab) in the settings of frontline treatment in cHL.

## Methods

### Search strategy and study eligibility

A comprehensive search was conducted via three major biomedical databases, including MEDLINE (via PubMed), EBSCOhost, and Cochrane Central Register of Controlled Trials (CENTRAL). Two main domains of keywords were used in a variety of combinations, including 1) PD-1 inhibitor(s), PD-1 inhibition, nivolumab, pembrolizumab and 2) Hodgkin(‘s) lymphoma, Hodgkin(‘s) disease. Searches were limited to full-text original data published in the English language only. Studies published from the moment of inception to September 15, 2025, were screened. An additional update for the literature search was done for studies published between September 15, 2025, and May 12, 2026. All references were additionally screened for potential eligible records. Articles presenting secondary data (i.e., commentaries and editorials, reviews and meta-analyses) were excluded.

The present systemic review was prepared based on the principles outlined in Preferred Reporting Items for Systematic Reviews and Meta-Analysis (PRISMA) (16).

### Inclusion and exclusion criteria

Prospective clinical trials were considered for the final review. To be eligible for the analysis, publications should have reported two or more predefined endpoints. Studies must have included patients with an established diagnosis of Hodgkin lymphoma who did not receive previous treatment for the disease but underwent frontline treatment with PD-1 inhibitor alone or in combination with other systemic therapy. Retrospective or observational studies, case reports or series, and preclinical studies were excluded.

### Outcomes

Primary outcomes for clinical efficacy were complete response rate (CRR), overall response rate (ORR), OS, and PFS. Grade ≥3 adverse events (AEs) were used for clinical safety. Studies were considered eligible if they reported two or more predefined clinical outcomes.

### Data extraction and quality assessment

Two reviewers (MG and DB) independently performed data extraction and quality assessment for each selected study. Discrepancies between reviewers were resolved by the third author (AO). Baseline characteristics of study populations as well as study details pertaining investigated treatment regimen were collected. The following items were extracted: registration number of the trial, study design and country, investigated treatment regimen with details, study sample size, comparison treatment (if any), stage of disease, Eastern Cooperative Oncology Group (ECOG) performance status, median age, median follow-up, additional treatments (if any), ORR, CR, PFS, OS, and grade ≥3 AEs.

### Risk of bias assessment

To assess the risk of bias and quality of selected studies, Cochrane risk-of-bias tool for randomized trials (RoB 2) was utilized for two randomized controlled trials and Methodological index for non-randomized studies (MINORs) for the rest of the clinical trials included (17, 18). Two reviewers (MG and AO) carried out evaluations. Three gradings were used for the evaluation: low, unclear, and high risks of bias. Discrepancies between reviewers were resolved by the third author (AG).

### Statistical analysis

For the clinical efficacy assessment, pooled CRR, ORR, 2-year OS, and 2-year PFS were analyzed using 95% confidence interval (CI). For the clinical safety evaluation, grade ≥3 AEs with 95% CI were calculated. For the study heterogeneity assessment, Cochran’s Q test (p-value <0.05) and I^2^ statistic (>50%) were utilized along with the random effects model. Where necessary, raw percentages were converted to proportions and multiplied by the study sample size to estimate event counts. A meta-analysis of proportions was performed using the meta prop function with a logit transformation (PLOGIT), inverse-variance method, and a random-effects model employing the DerSimonian-Laird estimator and Hartung-Knapp adjustment for a more conservative CI. Forest plots were generated to visualize pooled data with associated 95% CIs. Subgroup and sensitivity analyses were performed to identify the source of heterogeneity as well as to assess the optimal treatment regimen for frontline cHL according to the investigated drugs and features of the study population. A common effect model was used otherwise. Z-test was applied to assess differences between subgroups. For statistical significance, a two-tailed p-value of <0.05 was considered. R software (version 4.3.0) was used to conduct statistical analysis. For publication bias assessment, funnel plot asymmetry/linear regression/Begg’s test were utilized. If no asymmetry was demonstrated, no significant publication bias was assumed, which was then confirmed by the Egger test.

## Results

### Study selection and characteristics

**Figure 1:**
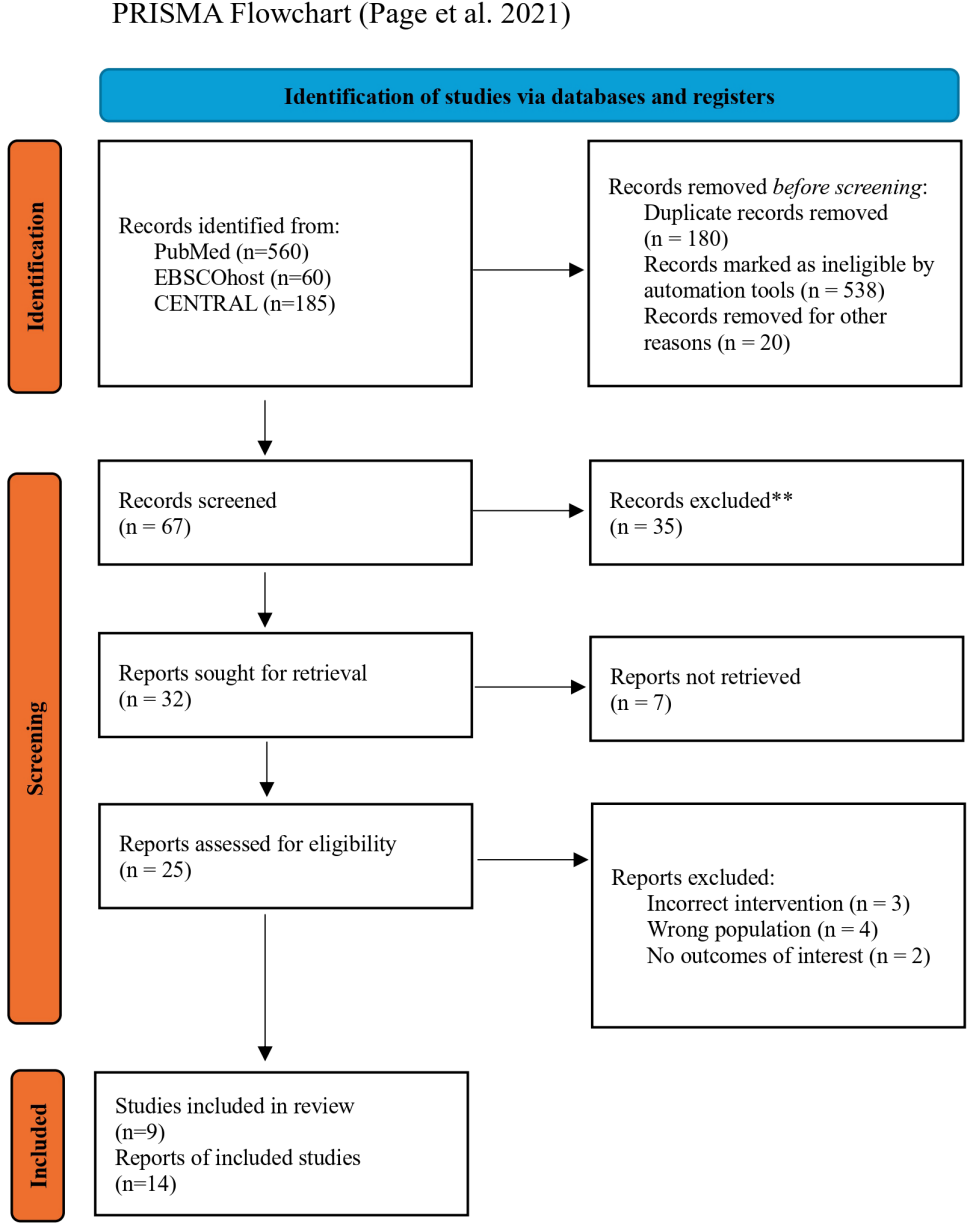
PRISMA flowchart [Page et al. (16)].

Our screening identified 9 multicenter trials reported in 13 publications with a total of 880 patients (**Figure 1**). Eight trials were Phase 2, while one was Phase 3. Four studies were conducted in the USA, three in the USA and Canada, one in Germany, and one internationally. Six trials were single-arm studies, and three were double-arm: one non-randomized and two randomized. Two trials investigated nivolumab in combination with BV, two trials studied pembrolizumab with AVD, four trials studied nivolumab with AVD, and one more trial investigated BV with nivolumab, doxorubicin, and dacarbazine. All but three trials (two studies in nivolumab with AVD and one study in pembrolizumab with AVD) utilized concurrent use of combination treatment. Six studies included patients with ECOG of 0-1, two had 0-2, and one study did not report. Five trials included patients of any disease stage, one trial studied patients with early disease (Stage I and II) but unfavorable prognosis, while the other three trials included patients with early (Stage I and II) but bulky disease as well as those with advanced disease (Stage III and IV). About 23% of all patients had early-stage disease (stage I and II). Forty-seven percent were female, the median weighted age of study participants was 33.8 years (range 27 to 72), while the median follow-up period of the study was 28 months (range: 11.1 to 51.6). The average number of immunotherapy treatment cycles (with nivolumab or pembrolizumab) was 7, ranging from 3 to 10. No significant variability in dose or regimen structure was observed. CR and ORR were reported in five trials, while PFS, OS, and grade ≥3 AEs were reported in all nine studies (**Table 1**; **Supplementray Table S1**). Selected endpoints (PFS, OS, CRR, ORR, grade ≥3 AEs) were either primary or secondary outcomes predetermined in the study protocols of each trial included in this meta-analysis. Responses in all trials were based on the Lugano Classification Revised Staging System for nodal non-Hodgkin lymphoma and cHL (2014 Lugano classification), except Ramchandren et al. (28) and Brockelmann et al. (27), where the 2007 International Working Group response criteria were utilized, as the study was designed earlier. In all studies, AEs were defined and reported in accordance with the National Cancer Institute Common Terminology Criteria for Adverse Events (NCI CTCAE).

**Table 1:** Baseline characteristics of patients and reported outcomes from all studies (n=9).

Study ID	Regimen	Sample size (n)	Stage of disease	Median age, years (range)	Female	Median follow-up, months	PFS (95% CI)	CRR (95% CI)	ORR (95% CI)	Grade ≥3 IRAE	Grade ≥3 AE	2-year PFS	2-year OS
Cheson 2020 NCT02758717	BV+N	46	Eary unfavorable (34%), advanced (66%)	71.5 (IQR 64-77)	46%	21.2 (IQR 15.6-29.9)	70% 18.3 months	N/A	61% (45-75)	N/A	80%	70%	94%
Lee 2025 NCT03646123	BV+N+AD	57	Early unfavorable (30%), advanced (69%)	35 (19-78)	47%	24.2	88% (75.7-94.6)	88% (76.3-94.9)	93% (83-98.1)	14%	14%	N/A	93%
Friedberg 2024 NCT01716806	BV+N	21	Early (19%), advanced (79%)	72 (60–88)​​	29%	51.6 (range: 1–72)	not reached	67% (43.0-85.4)	86% (63.7-97)	76%	76%	65%	85%
Herrera 2024 NCT03907488	N+AVD	496​	Advanced	27.6 (12.0–83.7)	44%	25.2 (range: 0–50.4)​	92% (89−94)	N/A	N/A	13%	29%	92%	99%
Torka 2024 NCT03033914	N+AVD	40	Early (22%) Advanced (78%)	66 (60-78)	45%	49 (range: 17-75)	79% (66-95)	N/A	N/A	10%	50%	86%	97%
Brockelmann 2022 NCT03004833	N+AVD	109	Early unfavorable	27 (18-60)	65%	41	99% (97-100)	98%	N/A	N/A	9%	99%	100%
Ramchandren 2019 NCT02181738	N+AVD	51	Early unfavorable (19%), advanced (81%)	37 (18–87)	37%	11.1 (range: 1.2–16.4)	92% (80-97) 9-months	80% (67-90)	84% (71-93)	10%	59%	N/A	N/A
Allen 2021 NCT03226249	P+AVD	30	Early unfavorable (40%), advanced (60%​)	29 (21–77)	63%	33.1 (range: 26.0-43.0)	100%	N/A	N/A	10%	23%	100%	100%
Lynch 2022 NCT03331341	P+AVD	30	Early (40%); advanced (60%)	33 (18–69)	60%	25.2	97%	90%	100%	13%	50%	97%	100%

BV+N(+AD), brentuximab vedotin + nivolumab (+doxorubicin + dacarbazine); CI, confidence interval; IRAE, immune-related adverse events; N+AVD, nivolumab + doxorubicin + vinblastine + dacarbazine; N/A, not available; OS, overall survival; P+AVD, pembrolizumab + doxorubicin + vinblastine + dacarbazine; PFS, progression-free survival.

### Efficacy analysis

**Figure 2:**
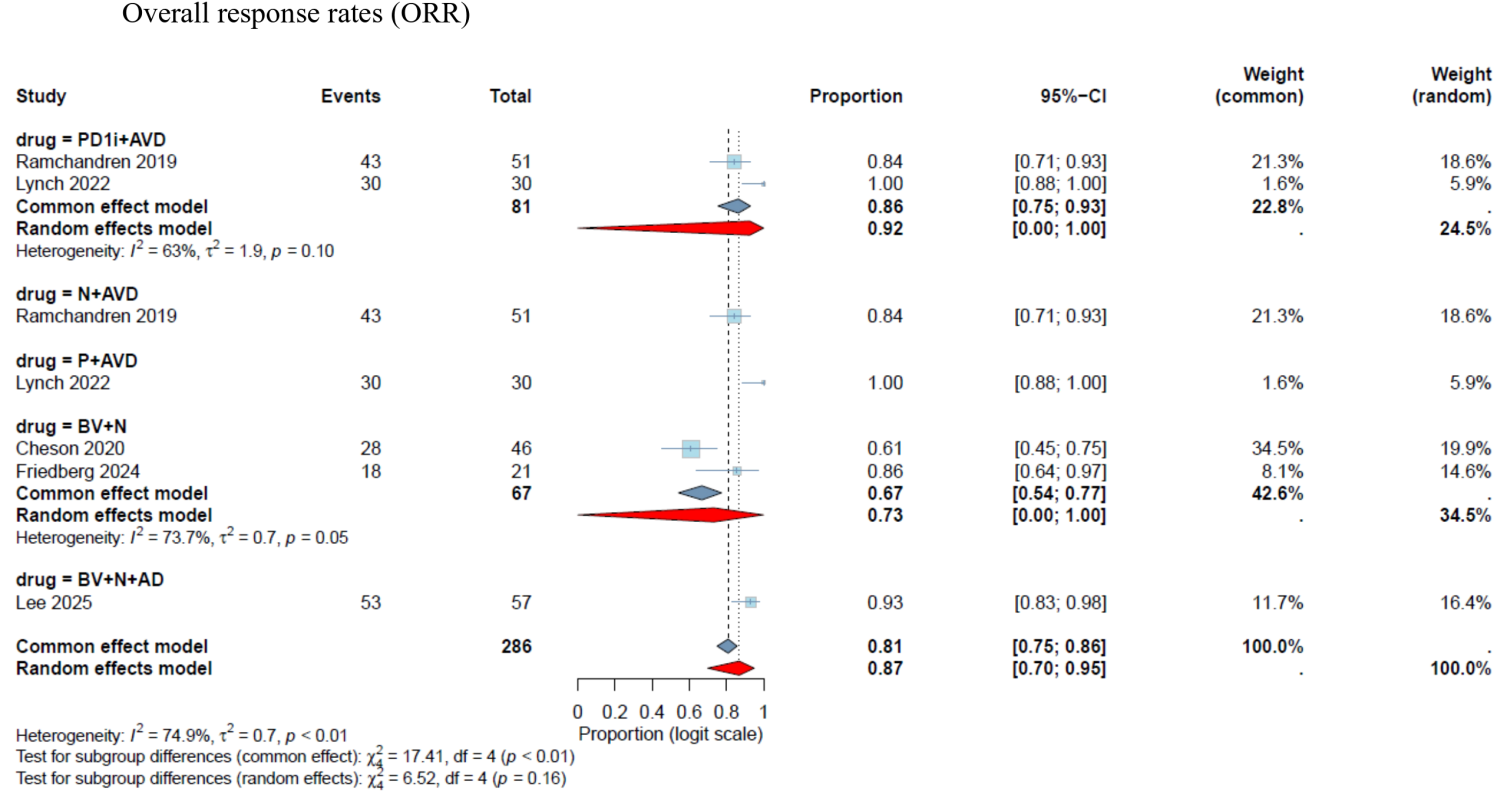
Overall response rates (ORR).

**Figure 3:**
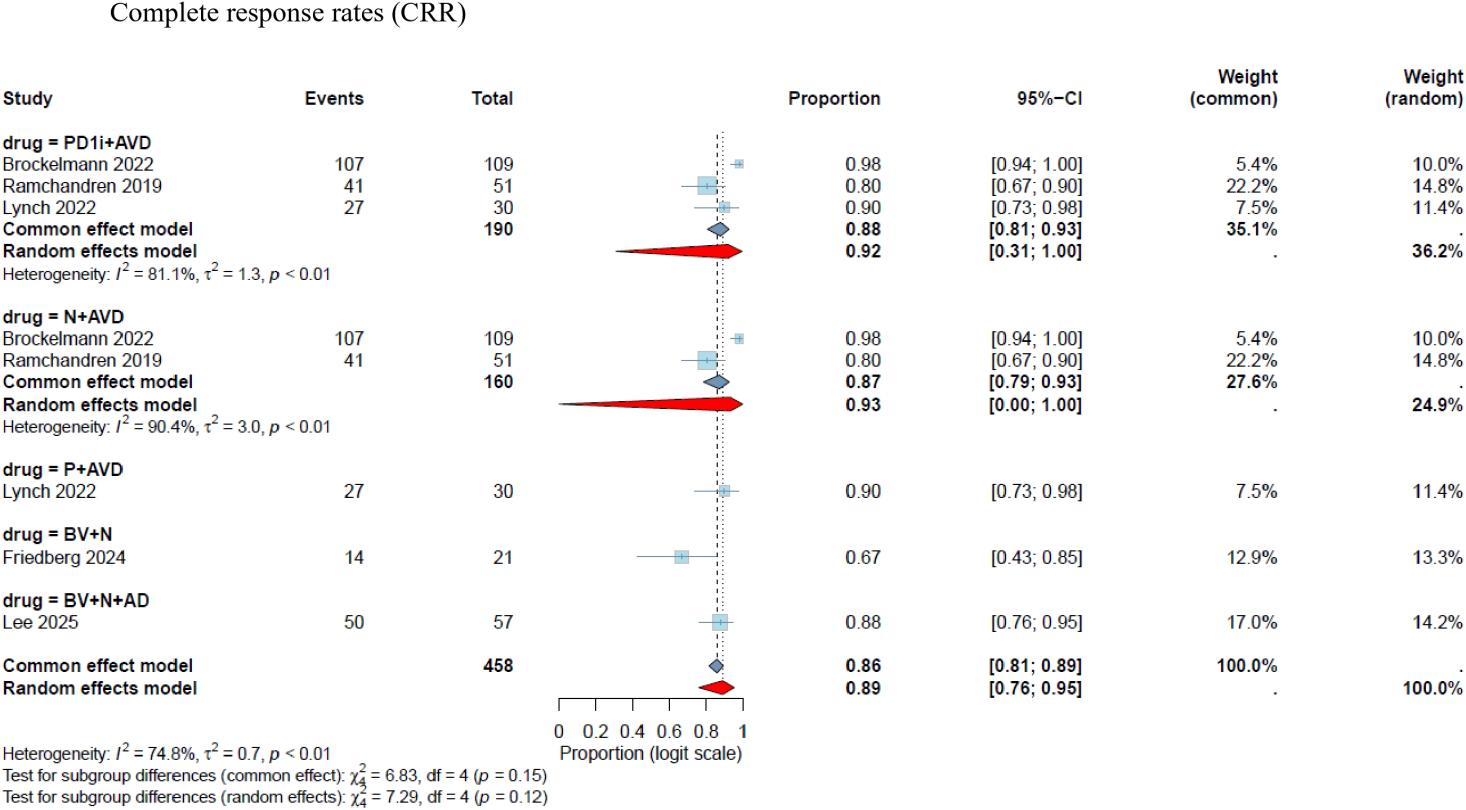
Complete response rates (CRR).

For the efficacy analysis of PD-1 inhibitors in frontline cHL, ORR, CRR, 2-year PFS, and 2-year OS were utilized as the main indicators. For ORR, only 5 trials reported data for a total of 205 patients, demonstrating pooled ORR of 86% (95% CI, 59%-96%; heterogeneity: I^2^ = 79.7%, r^2^ = 0.9, p<0.01) (**Figure 2**). For CRR, only 5 trials reported data for a total of 268 patients, resulting in a pooled CRR of 87% (95% CI, 62%-97%; heterogeneity: I^2^ = 76.0%, r^2^ = 0.7, p<0.01) (**Figure 1**). Two-year PFS was evaluated in 8 trials with a total of 859 patients, resulting in a pooled value of 90% (95% CI, 70%-97%; heterogeneity: I^2^ = 79.3%, r^2^ = 0.6, p<0.01) (**Figure 2**). Two-year OS was calculated for 8 trials with a total of 823 patients showing a pooled value of 97% (95% CI, 91%-99%; heterogeneity: I^2^ = 76.7%, r^2^ = 2.0, p<0.01) (**Figure 3**). Due to significant heterogeneity in all measurements, the random effects model was selected for the final report.

### Safety analysis

**Figure 4:**
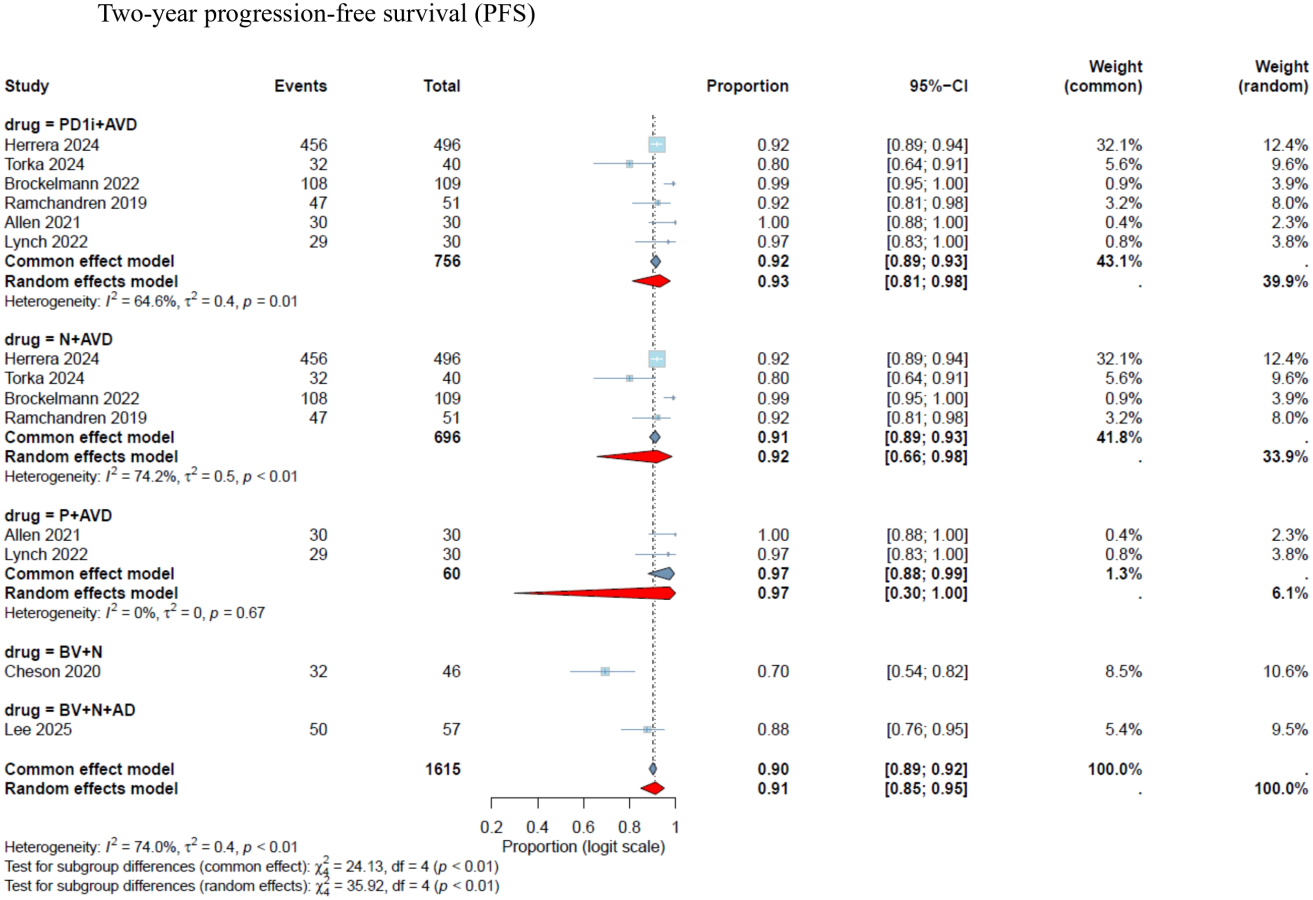
Two-year progression-free survival (PFS).

Grade ≥3 AEs was used as the sole measurement for the safety analysis of PD1 inhibitors in the treatment of cHL in frontline settings as reports within trials regarding grade 1 and 2 AE were either inconsistent or lacking. All 9 trials reported data on the safety profile of the regimen investigated, meaning that the entire sample (880 patients) of this systematic review was involved in the analysis. On average, 41% of patients (95% CI, 21%-64%) experienced grade ≥3 AEs in all treatment groups, when pooled data analysis was carried out. Random effects model was selected for the estimation of the safety analysis due to high heterogeneity observed in the calculation: I^2^ = 92.3%, r^2^ = 1, p<0.01 (**Figure 4**). Seven trials reported numerical data about immune-related AEs (IRAEs), where 17% (95% CI, 6%-38%; I^2^ = 83.3%, r^2^ = 0.7, p<0.01) experienced grade ≥3 severity, with the most common reported side effect being elevation in transaminases, accounting for 38.6% of all reported cases. This was followed by other gastrointestinal (GI) toxicities (13.1%), such as diarrhea, colitis, pancreatitis, and esophagitis; pneumonitis (3.5%) and overt hepatitis (3.5%) (**Supplementary Table 2**).

### Subgroup and sensitivity analyses

**Figure 5:**
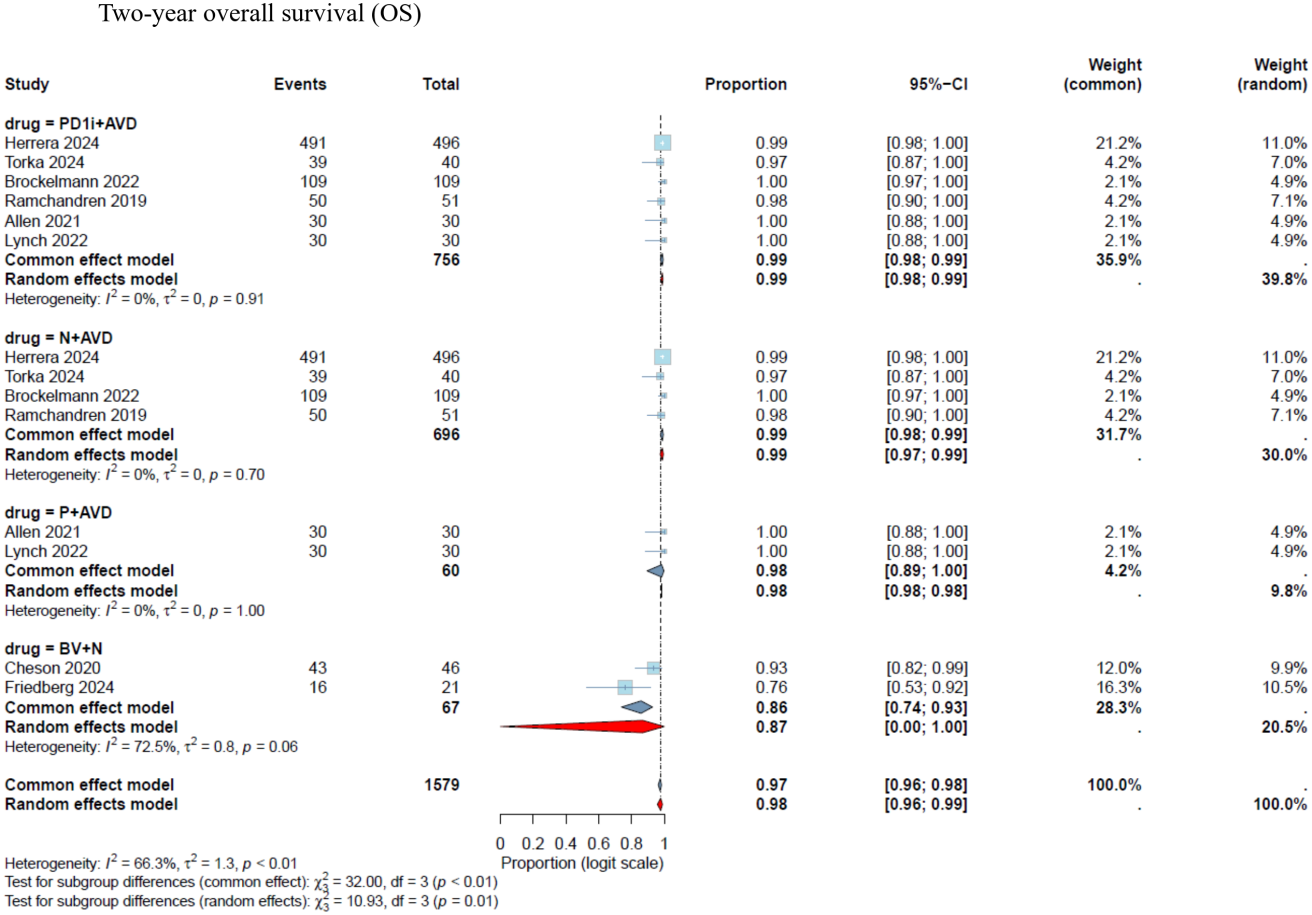
Two-year overall survival (OS).

**Figure 6:**
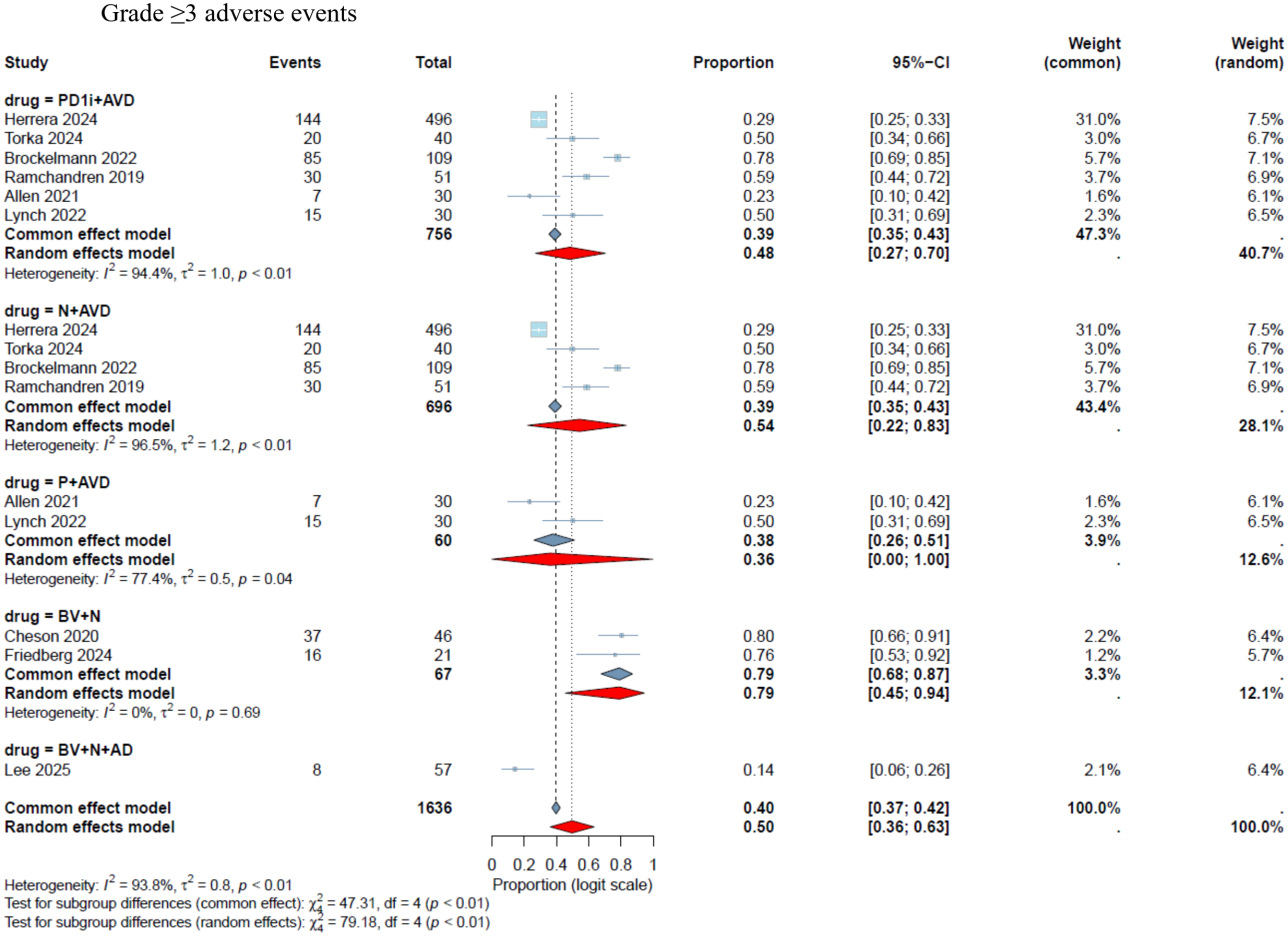
Grade ≥3 adverse events.

**Figure 7:**
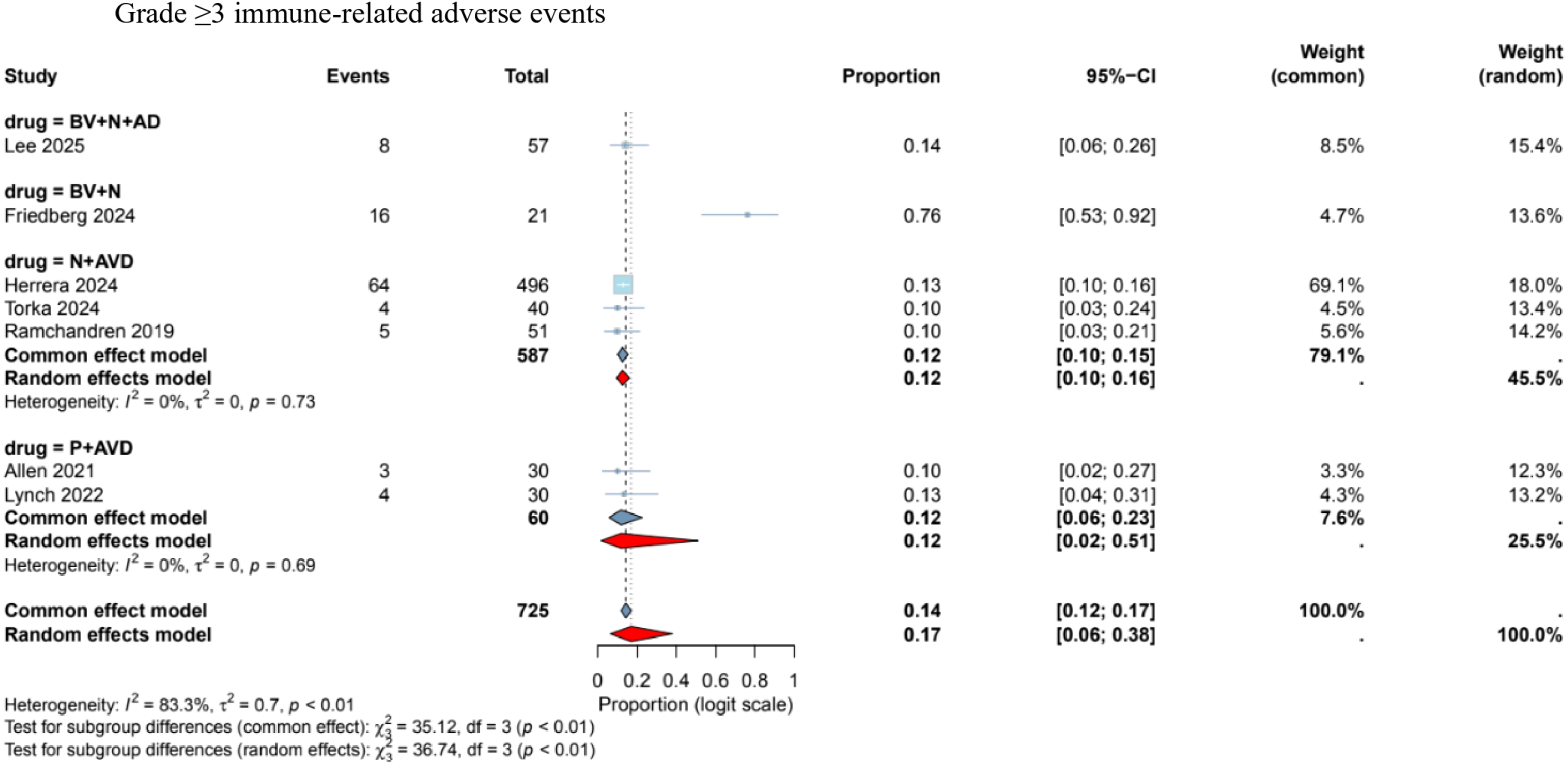
Grade ≥3 immune-related adverse events.

For the subgroup analysis, studies were divided according to the treatment received by patients (**Figures 2**–**7**). A combination of AVD with pembrolizumab or nivolumab showed comparable efficacy without statistically significant differences in all 4 indicators. For 2-year PFS, P+AVD group (n=60) had 97% (95% CI, 30%-100%, I2 = 0%, r2 = 0, p=0.67) vs N+AVD (n=696) with 92% (95% CI, 66%-98%, I^2^ = 74.2%, r^2^ = 0.5, p<0.01). For 2-year OS, P+AVD group (n=60) had 98% (95% CI, 88%-100%; I^2^ = 0%, r^2^ = 0, p=1.0) vs N+AVD group (n=696) had 99% (95% CI, 99%-100%; I^2^ = 0%, r^2^ = 0, p=0.70). Importantly, safety measured by grade ≥3 AEs was also similar between these two groups: P+AVD (n=60) 36% (I^2^ = 77.4%, r^2^ = 0.5, p<0.04) vs N+AVD (n=696) 33% (I^2^ = 93.2%, r^2^ = 0.8, p<0.01) with similar follow-up periods (29.1 vs 28 months) and mean age (33 vs 30.4 years old), respectively. Nivolumab with the BV regimen had comparably worse outcomes, although without statistical significance due to high heterogeneity. Pooled 2-year OS (n=67) was 87% (I^2^ = 72.5%, r^2^ = 0.8, p<0.06) and ORR was 73% (I^2^ = 73.7%, r^2^ = 0.7, p<0.05). Moreover, according to safety analysis, grade≥3 AEs were seen in 79% (95% CI, 45%-94%, I^2^ = 0%, r^2^ = 0, p<0.69) of 67 patients. Grade≥3 IRAE in N+BV group was available in one trial only, where it was 76% (95% CI, 53%-92%).

**Figure 8:**
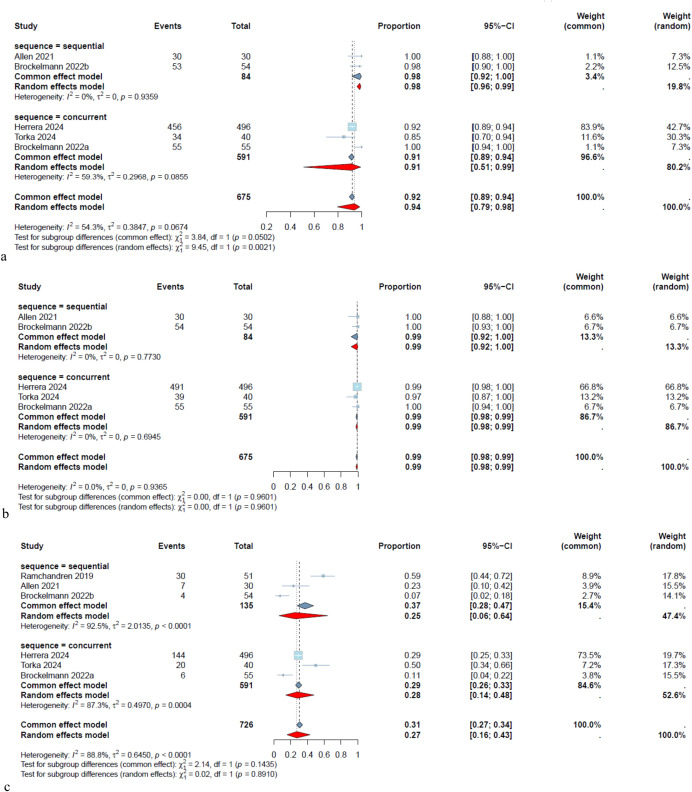
Subgroup analysis of comparison between sequential and concurrent administration of PD1 inhibitor plus AVD: progression-free survival **(a)**, overall survival **(b)**, grade ≥3 adverse events **(c)**.

We further analyzed patients receiving PD-1 inhibitors (both nivolumab and pembrolizumab) with AVD depending on the sequence of therapy received: concurrent vs sequential (**Figure 8**). Two studies utilized sequential administration of PD-1 inhibitors with chemotherapy, three studies did it concurrently, and one study had both treatments as separate arms. Pooled data analysis did not find any statistically significant difference in key outcomes of interest between sequential and concurrent administrations of treatments. Importantly, a trial with both arms also did not demonstrate any statistically significant differences between the two groups.

**Figure 9:**
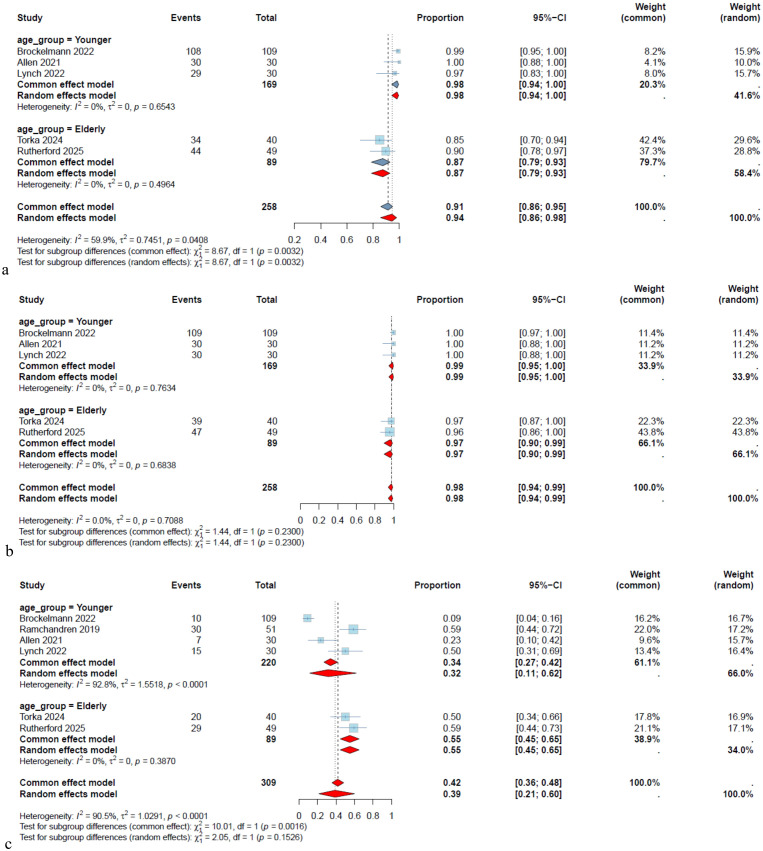
Subgroup analysis of comparison between sequential and concurrent administration of PD1 inhibitor plus AVD: progression-free survival **(a)**, overall survival **(b)**, grade ≥3 adverse events **(c)**.

Another sub-analysis was conducted to compare the efficacy of PD1 inhibitors with AVD based on patient age, dividing them into two main subgroups: younger (<65 years old) and older (≥65 years old) (**Figure 9**). Younger patients had better two-year PFS (98% (95% CI, 94%-100%) vs 87% (95% CI, 79%-93%), p<0.05) but similar two-year OS (99% (95% CI, 95%-100%) vs 97% (95% CI, 90-99%), p=0.7). Grade ≥3 AEs were less frequent among younger compared to older patients in the common effects model (34% (95% CI, 27%-42%) vs 55% (95% CI, 45%-65%), p=0.001 but the difference disappeared in the random effects model due to high heterogeneity in studies with a younger population.

No subgroup analysis was possible for the histological subtype and stage of the disease (early favorable, early unfavorable, or advanced) due to a lack of reported data. However, almost all individual trials (n=8) reported no differences in outcomes based on the stage of the disease. Similarly, we were not able to perform subgroup analysis by age group (elderly vs non-elderly patients) due to a lack of available data. However, several individual trials reported that older patients had worse outcomes while receiving the same treatment regimen.

The average number of cycles ranged significantly between clinical trials, depending on the regimen or the predominant stage of the disease in the studied population (**Supplementary Material**). For the PD1 inhibitor combination with AVD, the number of cycles ranged between 3 and 6, while for BV+N, it was either 8 or 10 cycles, respectively.

### Quality and bias assessment

For two randomized controlled trials, RoB 2 tool was utilized and demonstrated low risk of bias. For the other 7 non-randomized trials, MINORs was used, showing overall high quality of study methodology. Most of the studies score 15 out of 16 total points. One comparative study had a total score of 22 from 24 maximum. The assessment results are shown in **Supplementary Table 3**, **S4**.

## Discussion

The present meta-analysis of 9 multicenter clinical trials demonstrated that nivolumab and pembrolizumab can be incorporated in the frontline treatment of untreated cHL with high clinical efficacy and reasonable safety profile. Pooled data of 880 patients with a median age of 34 years old and a median follow-up of 31.2 months demonstrated ORR of 86% (95% CI, 59-96%), CRR of 87% (95% CI, 62-97%), 2-years PFS of 90% (95% CI, 78-96%), and OS of 97% (95% CI, 92-99%). Grade ≥3 AEs were seen in 41% (95% CI, 21-64%). In subgroup analyses, P+AVD and N+AVD were similarly safe and effective, while BV-N was linked with comparably worse tolerability. No differences were observed in terms of sequence of the treatment (concurrent vs sequential administration of PD1 inhibitors with chemotherapy) or stage of the disease (advanced vs early stage). In individual trials, elderly patients were likely to have worse efficacy and safety outcomes.

According to the current European clinical guidelines, management of early-stage cHL includes ABVD or eBEACOPP (reserved for fit patients with unfavorable disease), preferably with radiotherapy (19, 20). For favorable cHL in early stages, ABVD (2–3 cycles) combination with involved-site radiation (20–30 Gy) results in 87-99% PFS at 10-year follow-up (21–23). For unfavorable cHL, eBEACOPP-based strategy leads to >90% PFS at 10-year follow-up and is recommended for young (<60 years old) and/or fit patients (19, 20, 24). For advanced-stage cHL, the BrECADD regimen is recommended for fit and/or younger patients (<60 years old), while the combination of AVD with either BV or PD1 inhibitor is reserved for unfit or elderly patients (19, 20). BrECADD demonstrated 94.2% of 4-year PFS with a better toxicity profile compared to eBEACOPP, while BV+AVD was shown to result in 82% PFS at a 7-year follow-up based on ECHELON-1 trial results (13, 25). Pivotal SWOG S1826 study showed superiority of N+AVD compared to BV+AVD with 94% vs 86% 1-year PFS, although prior single-arm trials demonstrated comparable clinical efficacy of N+AVD and P+AVD (14, 26–30). PET guidance is applied in all stages for the optimal duration and number of cycles.

Clinical management guidelines from the National Comprehensive Cancer Network (NCCN), unlike the European Society of Medical Oncology (ESMO) and the Lymphoma Study Association (LYSA), recommend N+AVD with radiotherapy (30 Gy) in patients with early-stage unfavorable disease as an alternative to BV+AVD with radiotherapy (30 Gy) or BrECADD (19, 20, 31). The rationale stems from the results of the NIVAHL trial, where N+AVD led to 100% 2-year OS and 99% 2-year PFS in 109 patients with early-stage unfavorable cHL (26, 27, 32). In the present systematic review, 18% of all patients had early-stage disease (as reported in 6 trials). Although no data stratification was available for the stage of the disease, most trials did not report any correlations between outcomes and disease stage (29, 33, 34). We believe that future investigations should be focused on exploring the efficacy of regimens with PD-1 inhibitors in different stages of cHL.

As such, practice distinction exists between the US and Europe (particularly Germany) regarding limited adoption of eBEACOPP and later BrECADD, which primarily stems from different approach of early vs late intensification in the treatment of cHL. Early intensification relies on maximization of primary cure rates upfront, which in theory will help to avoid the need for salvage therapy in future. Conversely, late intensification models emphasize limitation of toxicity, while reserving high-dose chemotherapy for salvage therapy as well as autologous stem cell transplantation for future possible disease progression.

Optimal management of cHL in elderly patients remains an unmet need. Patients over 60 years old represent about 20% of all cases with cHL, yet account for 80% of all deaths. Moreover, this patient population has worse long-term survival outcomes and is prone to suffering more from treatment-related toxicities (11, 35–37). The latter leads to suboptimal therapy utilization, as only a fourth of elderly patients receive full-course chemotherapy (38). Three trials focused on elderly patients in our meta-analysis. Two trials showed that the BV+N regimen can result in 2-year PFS of 65-70% and 2-year OS of 85-94%, although at a cost of high frequency of grade ≥3 AEs: 76-80% (33, 39). Torka et al. demonstrated high clinical efficacy (2-year PFS of 86% and 2-year OS of 97%); however, half of the patients experienced grade ≥3 AEs (34). A subgroup analysis of the S1826 trial, including 99 patients aged 60 and older, showed 2-year PFS of 89% with N+AVD and 64% with BV+AVD (HR 0.24, 95% CI, 0.09-0.63) as well as 2-year OS of 96% with N+AVD versus 85% with BV+AVD (HR 0.16, 95% CI, 0.03-0.75) (40). Nonetheless, these outcomes appeared to be worse when compared to younger patients. Clinical guidelines by ESMO recommend stratifying the management of cHL by age. For patients >60 years old with early-stage unfavorable disease, 2 cycles of ABVD should be followed by 2 cycles of AVD, while for advanced age, 6 cycles of N+AVD are recommended (19). Prospective studies are needed to find balanced strategies in better management of cHL in the elderly population, taking into account comparably worse efficacy and tolerability of most of the treatments.

On average, 41% of patients (95% CI, 21-64%) experienced grade ≥3 AEs in all treatment groups. The most common side effects in both P+AVD and N+AVD were related to pancytopenia (primarily neutropenia followed by anemia), febrile neutropenia, and liver injury (elevated liver enzymes). In BV+N and BV+N+AD regimens, the most common problems were peripheral neuropathy (a known side effect of BV) as well as hepatotoxicity, an increase in lipase, and infections. Mucositis and end-organ damage were seen comparably rarer but almost in all treatment groups. Importantly, grade ≥3 IRAEs were observed in 17% (95% CI, 6-38%) of patients in the pooled analysis, with the only between-group difference seen in the BV+N group, which can be attributed to an older study population (mean age of 72 years) with mostly an advanced stage of the disease (79% of all patients). Elevation in liver enzymes accounted for more than a third of severe IRAEs cases, followed by GI toxicities (13.1%). PD1 inhibitors can lead to IRAEs in 20-30% of treated patients, while up to 5-10% experience severe grade ≥3 degree, which include endocrine, hepatic, dermatologic, pulmonary, and GI toxicities that can occur at any time point after exposure, with a median time of onset of 40 days, with <5% of fatality (41–44). The differences observed between our results and reported data in the literature can be attributed to patient-specific characteristics (e.g., prior comorbidities, age, or type and burden of primary malignancy), type of malignancy (most data stem from studies on solid tumors), and treatment-related risk factors (e.g., co-administration of chemotherapy, intensity, and lengths of therapy) (45, 46). Additionally, the overlap of organ damage manifestations makes the distinction between chemotherapy or radiotherapy-related AEs and IRAEs challenging. Most of the IRAEs cases are managed by cessation of PD1 inhibitors and administration of IV steroids or additional immunosuppressants (e.g., infliximab, mycophenolate mofetil, IVIG, or cyclophosphamide) (46).

No differences in OS, PFS, or AEs were observed in subgroup analysis stratified by the sequence of administration of PD-1 inhibitors and AVD regimen. Importantly, the study by Brockelmann et al. (27) also could not identify any differences between distinct administration sequences of the N+AVD regimen within their trial.

When stratified by age groups (elderly and younger adults), worse PFS (87% (95% CI, 79-93%) vs 98% (95% CI, 94-100%), p=0.0032) and AEs (32% (95% CI, 11-62%) vs 98% (95% CI, 45-65%), without statistical significance) were detected without contrasting OS. It is worth noting that these observations were earlier seen in a sub-analysis of the S1826 trial data.

P+AVD and N+AVD showed comparable efficacy in terms of survival outcomes (2-year PFS: 97% (95% CI, 88-99%) vs 92% (95% CI, 89-94%), 2-year OS: 98% (95% CI, 88%-100%) vs 99% (95% CI, 99-100%)) and safety profile (AEs: 36% vs 33%) with similar follow-up periods (29.1 vs 28 months) and mean age (33 vs 30.4 years old), respectively. Survival benefits were largely better compared to BV+N and BV+N-AD. The BV+N regimen had worse outcomes (PFS 80%, OS 87%, and AEs 79%), again likely due to a considerably older study population with higher percentage of advanced disease.

### Strengths and limitations

The present work has several important limitations. One of the major limitations of our meta-analysis is the high level of heterogeneity observed in the pooled data analysis, which restricts the generalizability of the findings. Another limitation is, only half of the trials included reported ORR and CRR, leading to possible undetected bias in our analysis. Additionally, not all trials were able to report adverse events attributed to PD1 inhibitors specifically, which requires caution in interpreting these findings. Furthermore, most of the included trials (6 out of 9) were single-arm trials, while two studies had a distinctly high sample size compared to the rest. However, these shortcomings are balanced by methodological strengths. The reported outcomes are based on a representative sample size of 880 patients and 9 multicenter Phase II and III clinical trials with low risk of bias per assessment. To the best of our knowledge, this is the first meta-analysis of clinical trials evaluating frontline treatment in patients with cHL, advancing the current knowledge based on the high level of evidence.

## Conclusion

Both pembrolizumab and nivolumab can be safely and efficiently incorporated into the frontline treatment of patients with cHL. This meta-analysis provides robust evidence regarding the clinical efficacy of nivolumab or pembrolizumab primarily in combination with AVD. Future studies should focus on investigating other combinations of PD1 inhibitors as well as specific patient populations (elderly vs young adults), histological subtype, and stage of the disease (early vs late).

## Data Availability

The original contributions presented in the study are included in the article/**Supplementary Material**. Further inquiries can be directed to the corresponding author.
