# Supplementary material for: Clinical efficacy and safety of pembrolizumab and nivolumab in frontline treatment for classical Hodgkin lymphoma: systematic review and meta-analysis of clinical trials
Source: Front Oncol. 2026 Jun 26;16:1865991. doi: 10.3389/fonc.2026.1865991 (PMC13349832; doi:10.3389/fonc.2026.1865991)
Supplement: Supplementary file 1 [file Table1.docx]

**Table S1**. Detailed characteristics of clinical trials

| **Study ID** | **Regimen investigated** | **Therapy sequence** | **Sample size (n)** | **Comparison treatment** | **ECOG** | **Stage of Disease** | **Treatment details** | **Additional treatments (radiotherapy)** | **Study design** | **Country** |
| --- | --- | --- | --- | --- | --- | --- | --- | --- | --- | --- |
| Cheson 2020 NCT02758717 | BV+N | concurrent | 46 | None | 0-2 | Stage I – 1  Stage II – 15  Stage III – 9  Stage IV – 21 | 8 cycles  (BV 1.8mg/kg (max 180mg) and N 3mg/kg) every 21 days | None | Phase II, single-arm, multicenter | USA |
| [Lee 2025 NCT03646123](https://doi.org/10.1182/blood.2024024681) | BV+N + AD | concurrent | 57 | None | 0-1 | Stage II – 18  Stage III – 10  Stage IV – 29 | 6 cycles  (BV 1.2mg/kg, N 240mg, doxorubicin 25mg/m^2^, dacarbazine 375mg/m^2^ on days 1&15 of 28-day cycle) | None | Phase II, single-arm, multicenter | USA |
| [Friedberg 2024 NCT01716806](https://doi.org/10.1182/blood.2022019536) | BV+N | concurrent | 21 | BV+DTIC | 0-1 | Stage I – 4  Stage II – 2  Stage III – 9  Stage IV – 7 | 10 cycles  BV (1.8mg/kg) and N (3mg/kg) every 21 days | None | Phase II, double-arm, non-randomized, multicenter | USA,  Canada |
| [Herrera 2024 NCT03907488](https://doi.org/10.1056/NEJMoa2405888) | N+AVD | concurrent | 496​ | BV+AVD | 0-2 | Stage III – 185  Stage IV – 302 | 6 cycles of N 240mg (adult) or 3 mg/kg (child) (max 240mg), and AVD (doxorubicin 25mg/m^2^, vinblastine 6mg/m^2^, dacarbazine 375mg/m^2^) on days 1&15 of 28-day cycle | Radiotherapy (30 Gy) was used for 7 patients (0,7%) | Phase III, double-arm, open-label, randomized, multicenter | USA,  Canada |
| [Torka 202](https://doi.org/10.1200/JCO-24-01278)4  NCT03033914 | N+AVD | concurrent | 40 | None | X | Stage I/II – 9  Stage III/IV – 21 | 6 cycles of AVD (doxorubicin 25mg/m², vinblastine 6 mg/m², dacarbazine 375mg/m²) plus N (240mg every 2 weeks) on days 1 &15 of 28-day cycle | None | Phase II, single-arm, multicenter | USA,  Canada |
| [Brockelmann 2022 NCT03004833](https://doi.org/10.1200/JCO.22.02355) | N+AVD | concurrent & sequential | 109  (concurrent, n=55 Sequential, n=54) | Concurrent vs sequential | 0–1 | Stage IA – 4  Stage IB– 1  Stage IIA – 82  Stage IIB – 92 | 4 cycles of N+AVD or  4 cycles of N then 2 cycles N+AVD, then 2 cycles of AVD,  (N 240mg, doxorubicin 25mg/m², vinblastine 6mg/m², dacarbazine 375mg/m² days 1&15 of 28-day cycle) | involved-site radiotherapy (30 Gy) | Phase II, double-arm, open-label, randomized, multicenter | Germany |
| [Ramchandren 2019](https://doi.org/10.1200/JCO.19.00315)  NCT02181738 | N+AVD | sequential | 51 | None | 0–1 | Stage II – 10  Stage III – 12  Stage IV – 19 | 4 cycles of N (240mg, every 2 weeks) then 6 cycles (every 2 weeks) of N+AVD (N 240mg, doxorubicin 25mg/m^2^, vinblastine 6 mg/m^2^, and dacarbazine 375mg/m^2^) | None | Phase II, single-arm, multicenter | USA,  Austria, Belgium, Canada, Czechia, Germany, Italy, Netherlands, Spain,  UK |
| Allen 2021  NCT03226249 | P+AVD | sequential | 30 | None | 0–1 | Stage IIA – 6  Stage IIB – 11  Stage IIIA –4  Stage IIIB – 1  Stage IVA – 6  Stage IVB – 7 | 3 cycles of P (200mg, every 21 days) then 4-6 cycles of AVD (doxorubicin 25mg/m^2^, vinblastine 6mg/m^2^, dacarbazine 375mg/m^2^, days 1&15 of a 28-day cycle) | None | Phase II, single-arm, multicenter | USA |
| [Lynch 2022](https://doi.org/10.1182/blood.2022019254)  NCT03331341 | P+AVD | concurrent | 30 | None | 0–1 | Stage I – 1  Stage II – 11  Stage III – 7  Stage IV – 11 | 6 cycles of P (200mg, every 21 days) and AVD (doxorubicin 25mg/m^2^, vinblastine 6mg/m^2^, dacarbazine 375mg/m^2^ on days 1&15 of 28-day cycle) | 3 patients (10%) with early-stage disease received preplanned consolidative radiotherapy | Phase II, single-arm, multicenter | USA |
| Abbreviation: AVD - Adriamycin (doxorubicin), Vinblastine, Dacarbazine; BV – Brentuximab Vedotin; DTIC – Dacarbazine; ECOG – Eastern Cooperative Oncology Group; Gy – Gray; N – nivolumab; P- pembrolizumab | | | | | | | | | | |

**Table S2**. Detailed characterization of immune-related adverse events

| **Author ID** | **Sample size** | **Immune-Related Adverse Events (Grade >3)** | **Description** |
| --- | --- | --- | --- |
| Lee 2025 | 57 | 14%, n=8 | Transaminitis (n=3)  Rash (n=2)  Colitis (n=1)  Hepatitis (n=1)  Pneumonitis (n=1) |
| Friedberg 2024 | 21 | 76%, n=16 | Not reported |
| Herrera 2024 | 496​ | 13%, n=64 | Transaminitis (n=34)  Diarrhea (n=8)  Rash (n=4)  Bilirubinemia (n=3)  Pancreatitis (n=3)  Pneumonitis (n=3)  Arthralgia (n=2)  Adrenal insufficiency (n=1)  Enterocolitis (n=1)  Esophagitis (n=1)  Hypothyroidism (n=1)  Lipase elevated (n=1)  Myositis (n=1)  Seizure (n=1) |
| Torka 2024 | 40 | 10%, n=4 | Hepatitis (n=1)  Acute interstitial nephritis (n=1)  Colitis (n=1)  Adrenal insufficiency (n=1) |
| Ramchandren 2019 | 51 | 10%, n=5 | Transaminitis (n=3)  Hepatitis (n=2) |
| Allen 2021 | 30 | 10%, n=3 | Transaminitis (n=2)  Bell’s palsy (n=1) |
| Lynch 2022 | 30 | 13%, n=4 | Transaminitis (n=2)  Rash (n=2) |

**Table S3.** Risk of bias assessment based on Revised Cochrane risk-of-bias tool for randomized trials (RoB 2)

| **Study ID** | Domain 1: Risk of bias arising from the randomization process | Domain 2: Risk of bias due to deviations from the intended interventions (effect of assignment to intervention) | Domain 3: Missing outcome data | Domain 4: Risk of bias in measurement of the outcome | Domain 5: Risk of bias in selection of the reported result | Overall risk of bias |
| --- | --- | --- | --- | --- | --- | --- |
| Herrera 2024 | Low | Low | Low | Low | Low | Low |
| Brockelmann 2022 | Low | Low | Low | Low | Low | Low |

**Table S4.** Quality of evidence assessment based on Methodological index for non-randomized studies (MINORs)

| **Study ID** | Clearly stated aim | Inclusion of consecutive patients | Prospective collection of data | Endpoints appropriate to the aim of the study | Unbiased assessment of the study endpoint | Follow-up period appropriate to the aim of the study | Loss to follow up <5% | Prospective calculation of study size | Adequate control group | Contemporary groups | Baseline equivalence of groups | Adequate statistical analyses | Total score |
| --- | --- | --- | --- | --- | --- | --- | --- | --- | --- | --- | --- | --- | --- |
| Allen 2021 | 2 | 2 | 2 | 2 | 1 | 2 | 2 | 2 | NA | NA | NA | NA | 15 |
| Cheson 2020 | 2 | 2 | 2 | 2 | 1 | 2 | 2 | 2 | NA | NA | NA | NA | 15 |
| Friedberg 2024 | 2 | 2 | 2 | 2 | 1 | 2 | 2 | 2 | 2 | 1 | 2 | 2 | 15 |
| Lee 2025 | 2 | 2 | 2 | 2 | 1 | 2 | 2 | 2 | NA | NA | NA | NA | 15 |
| Lynch 2022 | 2 | 2 | 2 | 2 | 1 | 2 | 2 | 2 | NA | NA | NA | NA | 16 |
| Ramchandran 2019 | 2 | 2 | 2 | 2 | 1 | 2 | 2 | 2 | NA | NA | NA | NA | 16 |
| Torka 2024 | 2 | 2 | 2 | 2 | 1 | 2 | 2 | 2 | NA | NA | NA | NA | 15 |
